# Comparing the responses of grain-fed feedlot cattle under moderate heat load and during subsequent recovery with those of feed-restricted thermoneutral counterparts: blood cells and inflammatory markers

**DOI:** 10.1007/s00484-023-02584-3

**Published:** 2023-12-13

**Authors:** G. Wijffels, M. L. Sullivan, S. Stockwell, S. Briscoe, R. Pearson, Y. Li, A. M. Macs, V. Sejian, R. McCulloch, J. C. W. Olm, J. Cawdell-Smith, J. B. Gaughan

**Affiliations:** 1 CSIRO Agriculture and Food, Queensland Bioscience Precinct, St Lucia, Qld 4067 Australia; 2 School of Agriculture and Food, The University of Queensland, Gatton, Qld 4343 Australia; 3 Rajiv Gandhi Institute of Veterinary Education and Research, Kurumbapet, Puducherry, 605009 India; 4 School of Veterinary Science, The University of Queensland, Gatton, Qld 4343 Australia

**Keywords:** Cattle, Cytokines, Endotoxin, Haptoglobin, HSP27, Hyperthermia, Neutrophils

## Abstract

Given the climate projections for livestock rearing regions globally, understanding the inflammatory status of livestock under various heat loads will be informative to animal welfare and management. A survey of plasma inflammatory markers was conducted, and blood leucocyte counts followed to investigate the capacity of the ~ 500 kg grain fed Black Angus steer to respond to and recover from a moderate heat load challenge. Two sequential cohorts of 12 steers were housed in climate-controlled rooms (CCR) for 18 days. A thermally challenged (TC) group (*n* = 2 × 6) experienced five consecutive periods: PreChallenge, Challenge, and Recovery within the CCR, and 40 days in outdoor pens (PENS and Late PENS). PreChallenge (5 days) and Recovery (7 days) delivered thermoneutral conditions, whereas in Challenge the TC steers experienced a diurnal temperature range of 28–35 °C. A feed-restricted thermoneutral (FRTN) treatment (*n* = 2 × 6) was run concurrently to differentiate between responses to reduced feed intake alone and moderate heat stress. Blood neutrophil counts were particularly sensitive to moderate heat load with higher numbers during Challlenge and in PENs. The plasma concentrations of TNFα and IL-1β were depressed in the TC group compared to the FRTN counterparts and remained so for 40 days after Challenge. Linear relationships of the concentrations of IL-1β, IL-10, and haptoglobin with rumen temperature or dry matter intake detected in the FRTN group were altered or absent in the TC group. The findings suggest significant impacts of moderate heat load on the inflammatory status of feedlot cattle.

**Supplementary Information:**

The online version contains supplementary material available at 10.1007/s00484-023-02584-3.

## Introduction

There is every reason to suspect that heat stress will provoke an inflammatory response in homeothermic animals. As blood is redirected to the periphery to assist with cooling, blood flow to the viscera and gastrointestinal tract (as well as the rumen) is reduced. In mildly, but transiently, heat-stressed sheep, Hales (1973a,b) reported the anticipated increased blood flows to some cutaneous sites and the nasal passages, but blood flows to the skeletal muscle, thyroid, kidney, spleen, rumen, and small intestine were reduced by 15–70%. Skeletal muscle and the rumen were most impacted. Reduced blood flows and splanchnic hypoxia were documented in transiently but severely heat stressed rats (attaining core temperatures of 41.5 °C) with the liver and intestine most affected (Hall et al. 1999). The interrelationships between tissue hypoxia and inflammation (Bartels et al. 2013) and the microvasculature and inflammation (Pober and Sessa 2015) are well described. Thus, blood flow and vascular changes resulting from hypoxia as a consequence of heat stress may initiate local and possibly systemic inflammatory responses.

A further consequence of reduced blood flows and hypoxia in the gastrointestinal tract are the deleterious impacts on gut integrity. Rodent models of heat stroke have revealed occurrences of ‘leaky gut’ whereby endotoxin from intestinal microbiota finds its way through the gut mucosa due to breakdown of the intestinal epithelium (Lambert et al. 2002; Lambert 2009). The concern is that endotoxin escapes into the systemic blood stream and triggers a potent and possibly unregulated inflammatory response. This is despite the capacity of the submucosal immune and inflammatory cell populations to neutralise toxins and the enormous capacity of the liver to remove endotoxin from the portal blood stream (Munford 2005).

The involvement of cytokines in heat stroke as evidenced by various rodent models and incidents of human heat stroke appeared to present a new angle upon which to monitor and assess heat stress in production animals. Reviews by Leon (2006), Heled et al. (2013), and Leon and Bouchama (2015) point to numerous studies with evidence of elevated levels of circulating cytokines (e.g., interleukin 1-β (IL-1β), tumour necrosis factor-α (TNFα), IL-6, and IL-10) and increased expression of these cytokines in various organs and tissues in response to heat stroke. However, heat stroke is a severe form of hyperthermia characterised by multi-organ failure and encephalopathy. Generally, the animal production industries are more concerned with increased heat load (i.e., compensable heat stress) and its consequences rather than heat stroke. If heat stroke occurs, it is often too late to intervene. The agency of the inflammatory response in heat stress in ruminants and other production species is not yet clear, especially in response to moderate heat load.

Many studies investigating aspects of the inflammatory responses in cattle have focused on dairy breeds and seasonal changes over the summer season. These studies detected adaptative and seasonal changes rather than responses to acute heat waves or spells of higher-than-average summer temperatures. As part of an intensive study on heat loads in the grain fed Black Angus feedlot steer, one of the hypotheses tested was that an inflammatory response would be induced under moderate heat load and subside as heat load abated. Changes to blood leucocyte populations and plasma concentrations of proinflammatory cytokines, haptoglobin (Hp), extracellular heat shock protein 27 (eHSP27), and endotoxin were monitored before, during, after moderate thermal load, and during the finishing phase in feedlot pens. These responses were compared to those of feed-restricted thermoneutral (FRTN) steers.

## Material and methods

### Animal experiments

The animal management including induction and transition to a feedlot finisher diet, and experiments in climate-controlled rooms (CCR) are described in detail by Sullivan et al. (2022). Experimental procedures were approved by The University of Queensland animal ethics committee (SAFS/210/13/MLA). In brief, three cohorts of 12 head of one-year-old Black Angus cattle were maintained in outdoor pens on a feedlot finisher diet at the Queensland Animal Science Precinct for 40 days in preparation for 18 days in the CCR and subsequent return to the outdoor pens for a further 40 days. Immediately before entry to the CCR, each cohort of 12 head was randomly allocated equally to two treatment groups (i.e., 2 treatments × 6 steers): thermally challenged (TC) and feed-restricted thermoneutral (FRTN). Cohorts were cycled through the CCR such that entry to the CCR for each cohort was approximately 21 days after the entry of the prior cohort.

**Fig. 1:**
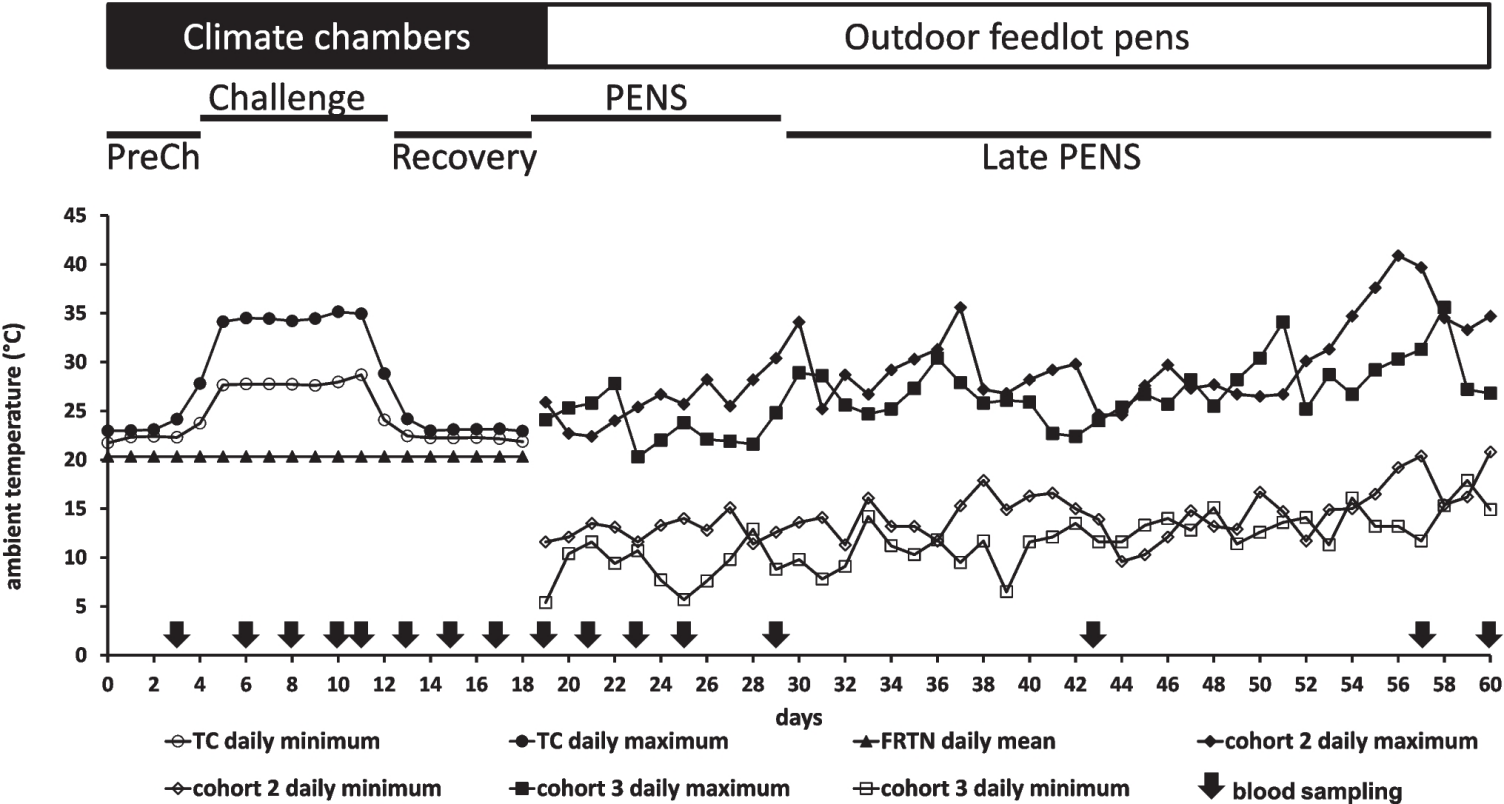
The climatic regimes imposed on the thermally challenged (TC) and feed-restricted thermoneutral (FRTN) groups. The daily ambient temperature range and duration of each period is given for the 60 days of the experiment. The PreChallenge (PreCh), Challenge, and Recovery periods (days 1–18) were conducted in climate-controlled rooms. The diurnal temperature range delivered to the TC group is presented as daily maximum and minimum temperatures for the PreChallenge, Challenge, and Recovery periods. The FRTN group was maintained in thermoneutral conditions and was feed-restricted throughout these three periods. On exit from the chambers (day 19), all animals were kept in outdoor feedlot pens till day 60. The daily maximum and minimum temperatures experienced by each of the two consecutive cohorts (cohorts 2 and 3) are given from days 19–60. Blood sampling regime is presented also

The 18 days in the CCR consisted of three different sequential periods for the TC treatment: PreChallenge, Challenge, and Recovery (Fig. 1). The day-to-day physiological and performance responses of all three cohorts during PreChallenge, Challenge, and Recovery are previously described (Sullivan et al. 2022). The current report focuses on the data collected from cohorts 2 and 3. [Cohort 1 was terminated after the Challenge period in the CCR to enable post-mortem tissue collection immediately after Challenge and thus did not contribute to data collected during Recovery, PENS, and Late PENS.]

During PreChallenge (4 days), the TC group was maintained in near constant thermoneutral conditions with the respective mean daily air temperature, %relative humidity (%RH), and Temperature-Humidity Index (THI) at 22.7 °C, 64%, and 70. The ambient temperature was cycled diurnally during the Challenge period (7 days) with the corresponding mean daily maximum air temperature, %RH, and THI at 34.5 °C, 58%, and 85.4; and the mean daily minimum air temperature, %RH, and THI were 27.9 °C, 36.4%, and 73.6 respectively (Fig. 1). Thermoneutral conditions were resumed in Recovery (7 days). The TC group were fed on an ad lib basis during the entire time in the CCR. Meantime, in a second independent room, the FRTN group remained in thermoneutral conditions for the entire 18 days with the respective mean daily air temperature, %RH, and THI at 20.3 °C, 67%, and 69. The FRTN steers were feed restricted during PreChallenge, Challenge, and Recovery, based on the feed intake and *feed offered* to a live weight matched pair within the TC group. In the *pair offered* approach, both members of the pair are offered the same amount of feed on the same day. However, the amount fed is based on the previous day’s intake of TC animal and topped up with an additional 20% by weight. During Challenge, this regime allowed the TC animal to feed ad lib and FRTN animal to be feed restricted. The purpose of using this approach was to avoid the deleterious consequences of abrupt reductions in feed intake in the FRTN animal (Sullivan et al. 2022). Water was always freely available. Mean liveweight of the cohort 2 and 3 steers on entry to the CCR was 516.8 ± 23.3 kg.

On exit from the CCR and for the final 40 days, the steers were held in a single outdoor feedlot pen and treated as a single group. This phase was split into two periods, PENS and Late PENS, during which time the animals were subjected to late winter to mid-spring conditions. Despite the two cohorts being about 3 weeks apart, they experienced very similar meteorological conditions in these two later periods (Fig. 1).

Detailed physiological and performance responses of TC and FRTN animals are given in Sullivan et al. (2022), and an aggregated form comparing the two treatment groups in each period is reported in Wijffels et al. (2022). In summary, mean dry matter intakes (DMI) were reduced 17–33% (from approximately 11 kg/head/day) during Challenge and Recovery, and mean live weights fell 15.6 and 18.4 kg for the FRTN and TC groups respectively over these two periods. The daily mean rectal temperature, respiration rates and water usage were all significantly increased in the TC group during Challenge, whereas these parameters gradually decreased in the FRTN group during this period, and the first three days of Recovery (Sullivan et al. 2022). By the end of the Recovery period, all mean physiological and performance measures for the two treatment groups had converged and returned to PreChallenge levels. By Late PENs, DMI and live weight had similarly and markedly increased in both groups (Wijffels et al. 2022).

### Blood sampling and leucocyte counts

The blood sampling schedule is displayed in Fig. 1. Blood was collected from all animals by jugular venepuncture from 0730 to 0800 h into 2 × 10 mL vacutainer tubes containing K_3_-EDTA and 2 × 10-mL vacutainer tubes containing lithium heparin (BD, Franklin Lakes, NJ). A single 10-mL K_3_-EDTA tube from each animal on each blood collection day was taken to Veterinary Laboratory Services (The University of Queensland, Gatton, Queensland) within an hour of collection to obtain a total white blood cell (WBC) count and differential. Plasma was prepared from the remaining blood tubes using standard procedures. The plasma for each animal was aliquoted into several 2-mL vials within 2 h of blood collection, transported on dry ice, and stored at − 80 °C until required.

### Plasma assays

Concentrations of plasma cytokines IL-1β, IFNγ, IL-10, and TNFα, eHSP27, and Hp were determined using sandwich enzyme-linked immunosorbent assays (ELISA). Assay plates (384 well, Perkin Elmer, MA) were coated overnight with the relevant capture antibody in sodium carbonate coating buffer (0.1 M Na_2_CO_3_ pH 9.6) at 4 °C. Recombinant protein standards were serially diluted to appropriate concentrations in pooled heat-inactivated clarified foetal bovine serum. The pooled calf serum was shown to have low background for all the target proteins. Plasma samples (EDTA) and standards were then diluted in Tris-buffered saline containing Tween 20 (TBST: 50 mM Tris, 150 mM NaCl (pH 7.6), and 0.1% Tween 20). Standards (prepared in the pooled calf serum) and neat plasma samples were diluted 1:50 in TBST for the IL-1β and TNFα assays, 1:2 for the IFNγ and IL-10 assays, 1:20 for the HSP27 assay, and 1:2500 for the Hp assay.

All reagents were delivered using an EpMotion 5075 liquid handling robot. The concentrations of capture and detection antibodies are given in Supplementary Table 1. Plates were manually washed 4 times between each step in TBST. The coated assay plates were blocked for 30 min in 2% skim milk powder in TBST. The diluted samples (in duplicate) and standards (in quadruplicate) were added to the plates and incubated for 1 h at room temperature (RT). For the Hp and IL-10 assays, diluted samples were added in triplicate. Detection antibodies (in TBST) were added to all wells and incubated for 30 min at RT; amplification (30 min, RT) was performed for the cytokines and HSP27 using Streptavidin/HRP (Pierce 1:20,000). Results were visualised with tetramethylbenzidine (Biorad Core +) for IL-10, and TNFα and the reaction stopped with 2% sulphuric acid. IL-1β and IFNγ assays were measured with QuantaBlu (a fluorogenic substrate, Thermo Fisher). The haptoglobin detection antibody was labelled with peroxidase; thus, no amplification step was required. The presence of Hp was detected using 1 mM 2,2′-azinobis-(3-ethylbenzothiazoline-6-sulfonic acid) in 0.1 M citric acid (pH 4.0) and 0.01% hydrogen peroxide. The assay development was measured on a Spectramax M3 plate reader (Biostrategy). Assay performances are given in Supplementary Table 2.

### LPS assays

Lipopolysaccharide concentrations in plasma samples were measured using the QCL-1000 Endotoxin assay kit (Lonza) according to the manufacturer’s instructions. Plasma samples were diluted 1:40 in 10 mM Tris prepared in endotoxin free water (Lonza) and inactivated at 70 °C for 20 min. Supplied standards, lysate, and chromogen solutions were all prepared using endotoxin free water and delivered using the EpMotion 5075 robot. Diluted and inactivated plasma samples and prepared standards (50 µL) were added to sterile 96-well plates (Nunc). Lysate (50 µL) was added to all wells for 10 min at 37 °C followed directly by Chromogen (100 µL) for 6 min at 37 °C. The reaction was stopped using 10% SDS, and the reaction product was visualised at 405 nm by the Spectramax M3 plate reader.

### Statistical analysis

The plasma concentrations obtained for TNFα, IFNγ, IL-10, Hp, and eHSP27 showed log-fold inter-animal variability. The values were log-transformed to better meet assumptions of ANOVA*.* A total of seven plasma constituents (IL-1β(log10), IFNγ(log10), IL-10(log10), Hp(log10), eHSP27(log2), TNFα and endotoxin), six leucocyte population counts (WBC, neutrophils, PBL, monocytes, eosinophils, and basophils), and the neutrophil: lymphocyte (NL) ratio were analysed. The statistical analysis procedure has been described in detail previously (Wijffels et al. 2022). In brief, a linear mixed model of analysis of variance (ANOVA) was applied to each individual parameter using the statistical program SAS (version 9.4, SAS Institute Inc., Cary, NC, USA; 2002–2012). The purpose was to evaluate if there was a significant difference in parameter concentrations between TC and FRTN animals within or across different time periods. In each analysis, the fixed effects assessed in the model included treatment (TC vs FRTN), cohort (2 vs 3), period (PreChallenge vs Challenge vs Recovery vs PENS vs Late PENS), and their two-way and three-way interactions. The effect due to different animal pairs was examined as a random effect. Period was fitted as a fixed effect rather than a covariate. Type 3 SS ANOVA and the estimated least-square mean (LSM) and standard error of mean (SEM) were produced for individual fixed effects and their interactions. The PROC PLM model (SAS) enabled assessment of the significance of all pair-wise comparisons amongst the individual fixed effects.

During the PreChallenge period, all animals regardless of the later treatment groupings were subjected to the same management, and no significant difference between TC and FRTN groups within PreChallenge period was observed using an initial ANOVA. Consequently, the animal data collected for the PreChallenge period were pooled across the two cohorts, and all animals were used to determine the PreChallenge period mean and SEM for individual variables. Discovery of relationships of the log-transformed daily mean concentrations of the cytokines, Hp, and eHSP27 well as daily mean endotoxin concentrations with the corresponding daily means of rumen temperature or daily mean DMI during the PreChallenge, Challenge, and Recovery periods (the 18 days in the CCR) was performed in Prism 9.0 (GraphPad Software, San Diego, CA). *P*-values less than 0.05 were considered significant. When the *p*-value fell into the 0.05–0.08 range (0.08 ≥ *p* ≥ 0.05), a trend towards significance was considered.

## Results

### Trajectories over the five periods (PreChallenge through to Late PENs)

#### Leucocyte counts

**Fig. 2:**
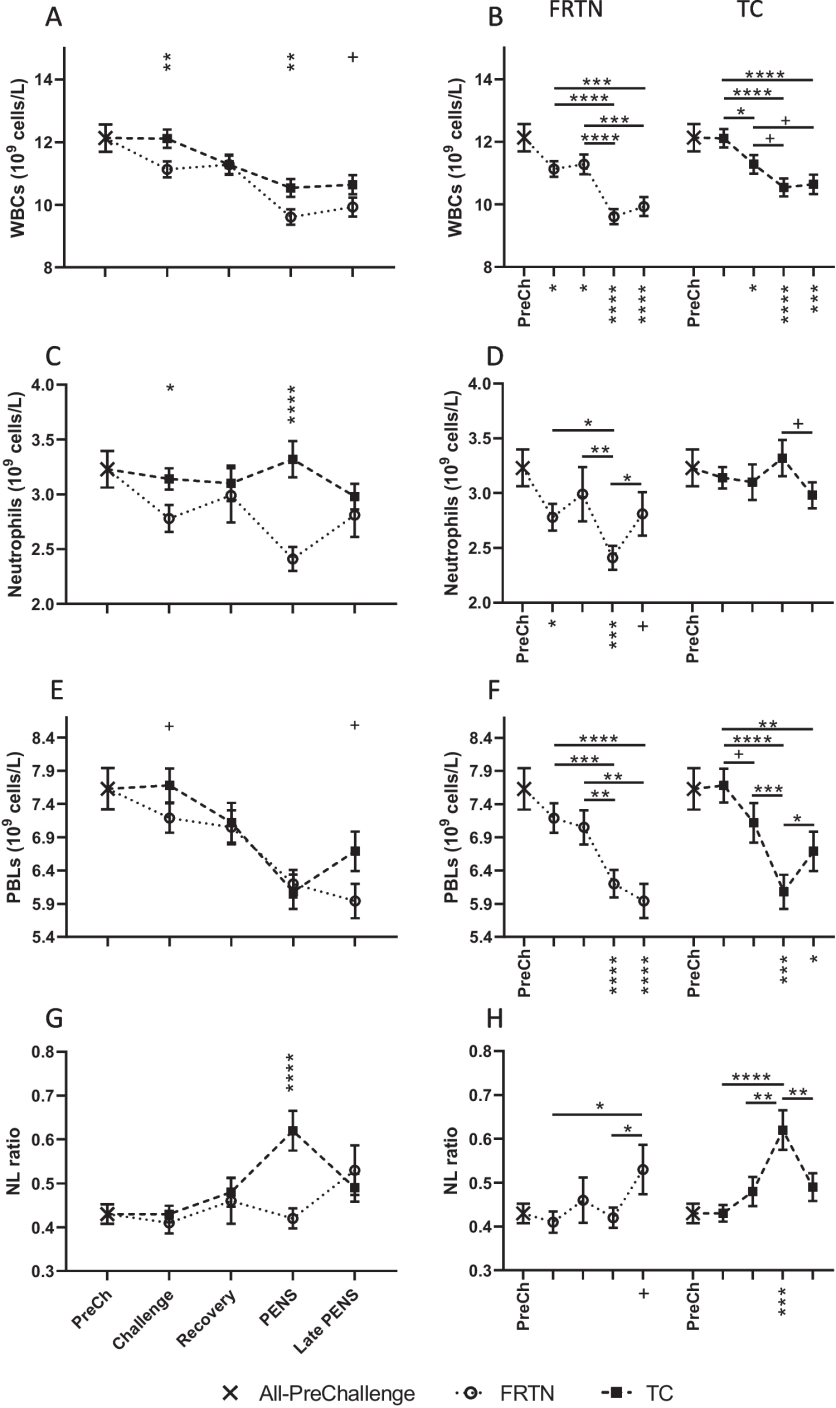
Changes in the counts of total white blood cells (WBC), neutrophils, peripheral blood lymphocytes (PBL), and the neutrophil:lymphocyte (NL) ratio during the five periods: PreChallenge, Challenge, Recovery, PENS, and Late PENS. FRTN animals were feed-restricted in thermoneutral conditions until PENS and Late PENS. The PreChallenge (PreCh) value in each graph is the mean (± SEM) of all animals during that period (denoted by X). **A, C, E, and G** Comparison of the mean values (± SEM) of the treatment groups for WBC, neutrophil and PBL counts respectively, and the NL ratio. Significant differences are indicated by the asterisks above the period means. **B, D, F, and H** Within group comparisons for each group for WBC, neutrophil, and PBL counts respectively and the NL ratio across the five periods (PreChallenge, Challenge, Recovery, PENS, and Late PENS: only PreCh is labelled on the *x*-axis). The asterisks under the *x*-axis indicate statistically significant difference with the PreChallenge mean. ^+^*p* < 0.1; ^*^p < 0.05; ^**^p < 0.01; ^***^p < 0.001; ^****^p < 0.0001

There was significant effect of period on the total white blood cell (WBC) counts (*p* < 0.0001) with a gradual but significant reduction (12–21%) over time observed in both groups relative to the PreChallenge mean count (Fig. 2A, B). There was also a significant treatment effect (*p* = 0.0017) with mean WBC counts higher in the TC group during Challenge (*p* = 0.0029) and in PENs (*p* = 0.0015) relative to the FRTN group. There was no significant interaction of treatment by period on mean WBC counts (*p* = 0.148). There was significant treatment effect on mean neutrophil count (*p* = 0.0010) and a treatment by period interaction (*p* = 0.0052). These predominantly manifested in a reasonably stable mean neutrophil count across the periods in the TC group and substantial fall in neutrophil count in the FRTN group during PENS. The PENS mean for FRTN group was 25–27% lower than the corresponding TC and PreChallenge means (Fig. 2C, D). There was no effect of period on neutrophil counts (*p* = 0.155).

In contrast, peripheral blood lymphocyte (PBL) counts displayed a significant effect of period (*p* < 0.0001), with no overall treatment effect (*p* = 0.107) or a treatment by period interaction (*p* = 0.244). The FRTN group saw a gradual fall in mean PBL counts as the trial progressed, so that the PBL counts during PENS and Late PENS were significantly lower than those of the preceding periods (Fig. 2E, F) and 12–22% reduced compared to the PreChallenge mean. The TC group showed slightly different dynamics with a slightly higher PBL count during Challenge (relative to the FRTN group), and a rebound in PBL count in Late PENS (Fig. 2E, F). The mean neutrophil: lymphocyte (NL) ratios revealed a trend towards an effect of treatment (*p* = 0.0925) and a significant effect of period (*p* = 0.0070). There was a significant treatment by period interaction (*p* = 0.0008) reflecting the comparatively high NL ratio of the TC group during PENS. The TC mean NL ratio in PENS was 44–48% elevated over the PreChallenge mean and corresponding FRTN mean (Fig. 2G, H).

**Fig. 3:**
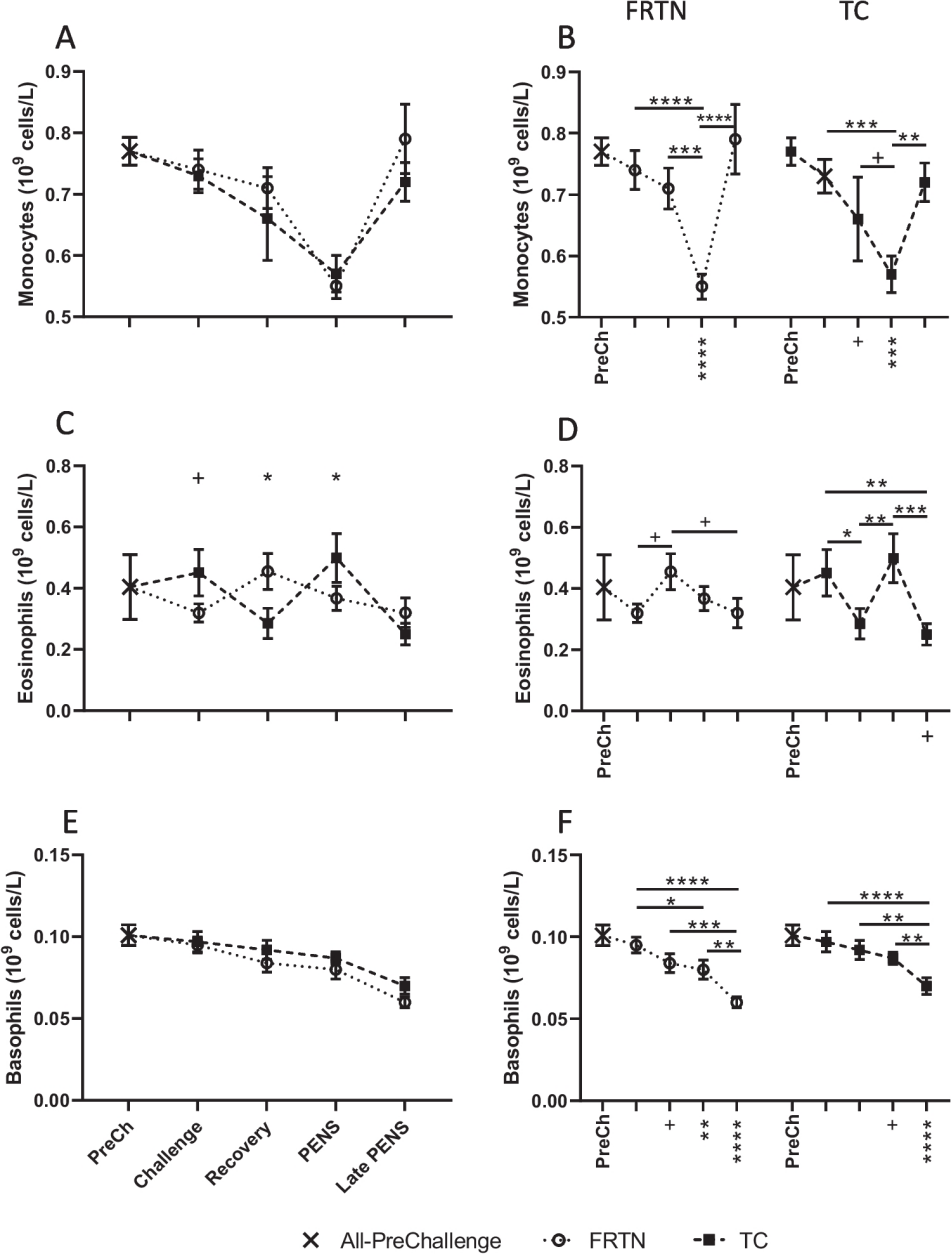
Changes in the counts of the minor leucocyte subpopulations during the five periods. The PreChallenge (PreCh) mean denoted by *X*. **A, C, and E** Comparison of the mean values (± SEM) of the groups for monocyte, eosinophil, and basophil counts respectively. Significant differences are indicated by the asterisks above the period means. **B, D, and F** Within group comparisons for each group for monocyte, eosinophil, and basophil counts respectively across the five periods (only PreCh is labelled on the *x*-axis). The asterisks under the *x*-axis indicate statistically significant difference with the PreChallenge mean. ^+^*p* < 0.1; ^*^p < 0.05; ^**^p < 0.01; ^***^p < 0.001; ^****^p < 0.0001

There was a significant effect of period on mean monocyte count (*p* < 0.0001) with both groups experiencing 26–29% reductions in mean monocyte counts relative to the PreChallenge mean (Fig. 3A, B). There was no overall effect of treatment (*p* = 0.300) nor a treatment by period interaction (*p* = 0.673). The mean monocyte counts were not different between the treatment groups at any point. There were no overall effects of treatment (*p* = 0.875) or period (*p* = 0.061) on mean eosinophil counts (Fig. 3C, D); however, there was an interaction of treatment and period (*p* = 0.0141). The mean eosinophil counts of the two groups showed different profiles during Challenge, Recovery, and PENS. The FRTN eosinophil counts were quite stable over the periods, whereas the counts for the TC group showed greater variation across the periods (Fig. 3D). Basophil counts were significantly affected by period (*p* < 0.0001) with decreasing counts over time but were unaffected by treatment (*p* = 0.131, Fig. 3E, F). The mean basophil counts in Late PENS were 30–40% reduced compared to the PreChallenge mean.

#### Cytokines

**Fig. 4:**
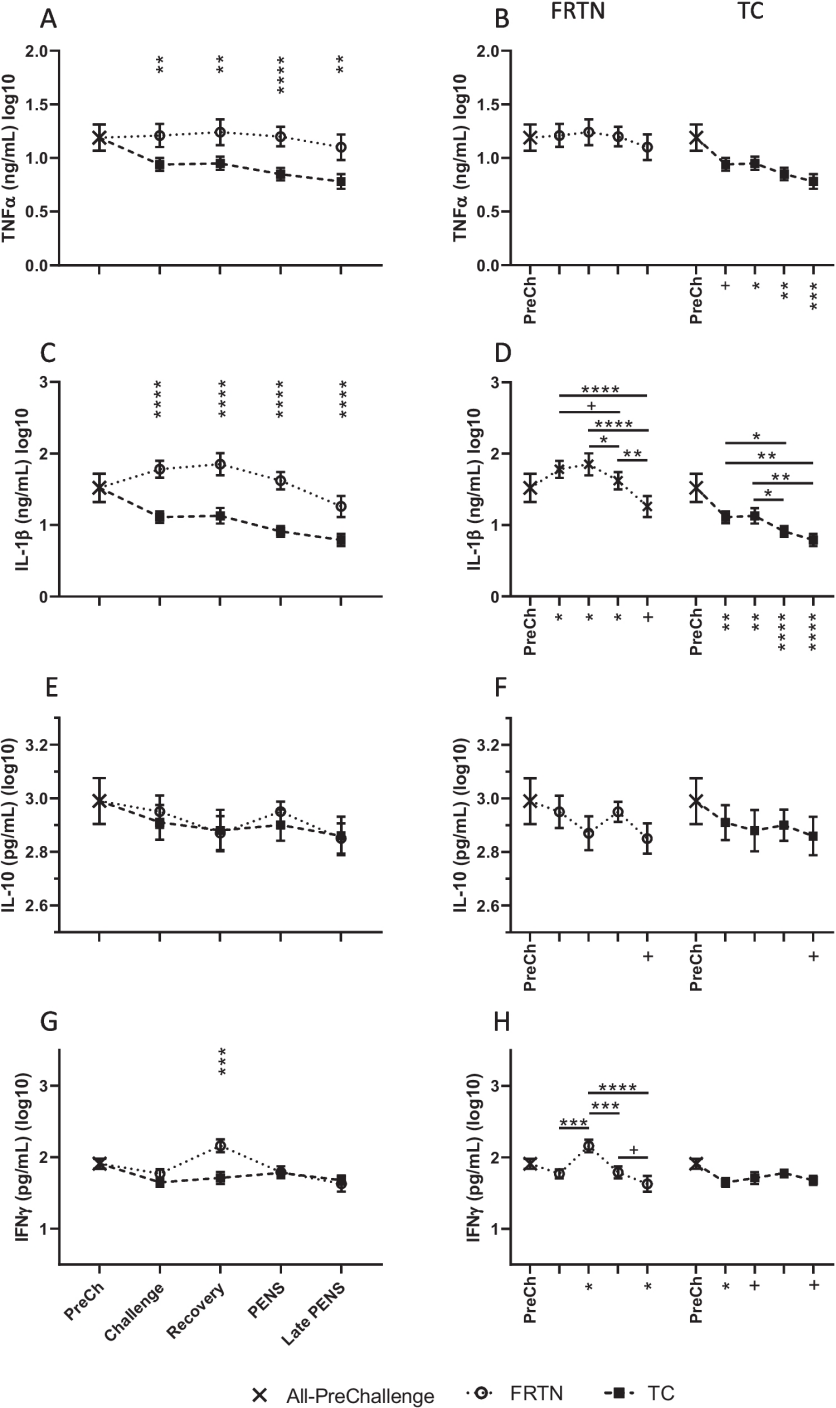
Changes in plasma cytokine concentrations during the five periods. The PreChallenge (PreCh) mean denoted by *X*. **A, C, E, and G** Comparison of the mean values (± SEM) of the groups for the log10 concentrations of TNFα, IL-1β, IL-10, and IFNγ respectively. Significant differences are indicated by the asterisks above the period means. **B, D, F, and H** Within group comparisons for each group for the log10 concentrations of TNFα, IL-1β, IL-10, and IFNγ respectively across the five periods (only PreCh is labelled on the *x*-axis). The asterisks under the *x*-axis indicate statistically significant difference with the PreChallenge mean. ^+^*p* < 0.1; ^*^p < 0.05; ^**^p < 0.01; ^***^p < 0.001; ^****^p < 0.0001

There was a significant effect of treatment (*p* < 0.0001) on mean TNFα(log10) concentration and an effect of period (*p* = 0.0476), but interaction between treatment and period was not evident (*p* = 0.258). The mean TNFα(log10) concentrations of the TC group were consistently and significantly lower than those of the FRTN group in all periods (Fig. 4A). The TC group means in Recovery, PENS, and Late PENS were significantly lower than the PreChallenge mean also (Fig. 4B). In contrast, mean TNFα(log10) concentrations of the FRTN group were stable throughout. Likewise, there was a significant effect of treatment on IL-1β(log10) concentration (*p* < 0.0001) with reduced mean IL-1β(log10) concentrations in the TC group compared to the FRTN group in all periods and relative to the PreChallenge mean (Fig. 4C, D). A significant effect of period (*p* < 0.0001) was detected also for both groups; Late PENS presented with the lowest IL-1β(log10) concentrations. There was a significant treatment by period interaction (*p* = 0.0005) on IL-1β concentrations also. The FRTN group exhibited higher mean IL-1β(log10) concentrations compared to the PreChallenge mean during Challenge, Recovery, and PENS, whilst the TC group experienced an immediate fall in mean IL-1β(log10) concentrations in those same periods (Fig. 4D).

In contrast, there was no evidence of any treatment or period effects on the mean IL-10(log10) concentrations (*p* = 0.641 and *p* = 0.112 respectively, Fig. 4E, F) reflecting the very stable levels of this cytokine in both groups. There was an overall effect of treatment (*p* = 0.0260) on IFNγ(log10) concentration, due mostly to the increased IFNγ (log10) concentration in the FRTN group in Recovery relative to the TC group and PreChallenge means (Fig. 4G, H). There was a significant effect of period on IFNγ(log10) concentrations (*p* = 0.0007) for both groups with a small reduction in concentration by Late PENS and a significant interaction between treatment and period (*p* = 0.0066).

#### Haptoglobin, eHSP27, and endotoxin

**Fig. 5:**
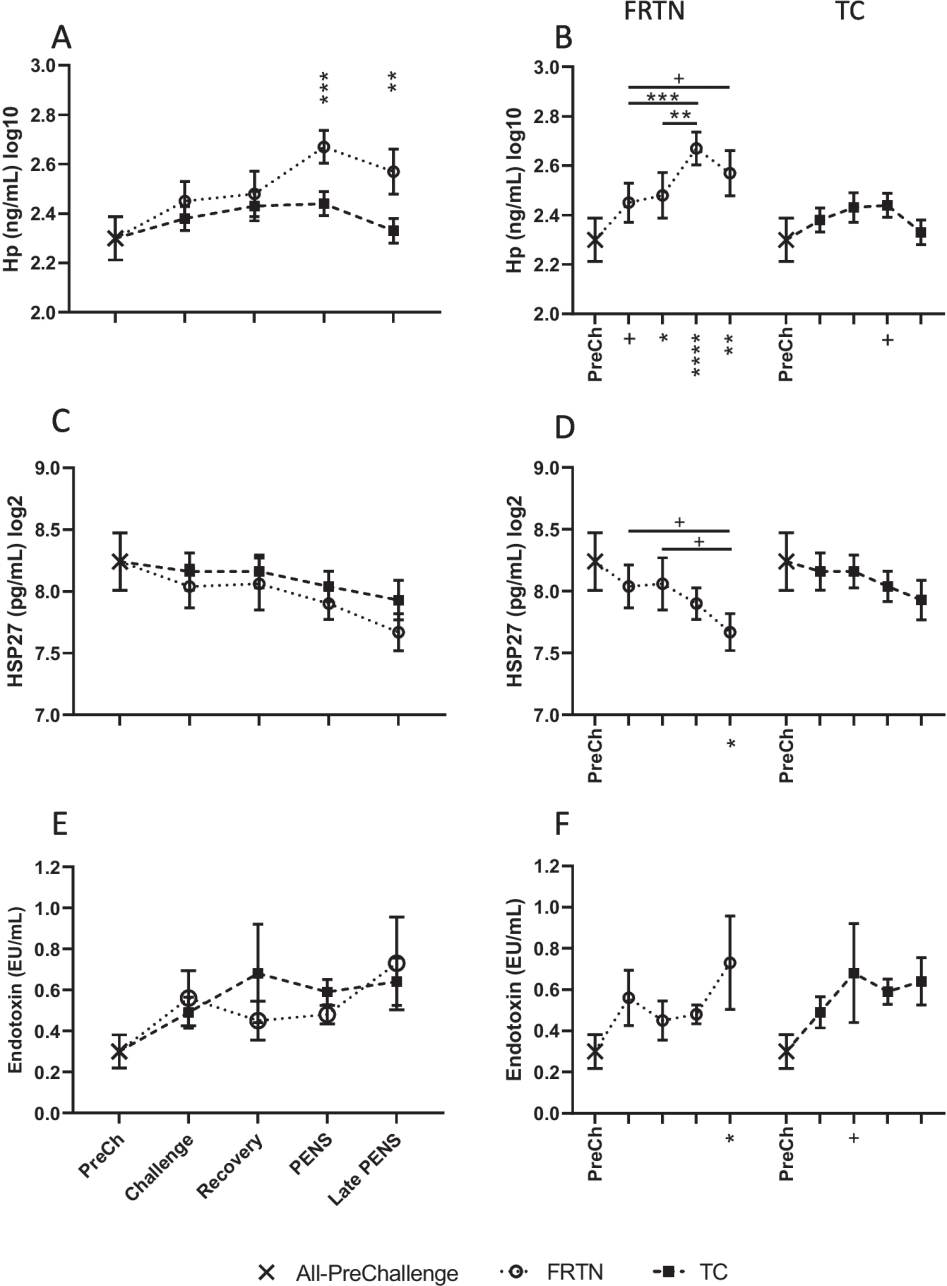
Changes in plasma haptoglobin (Hp), extracellular heat shock protein 27 (eHSP27), and endotoxin concentrations during the five periods. The PreChallenge (PreCh) mean denoted by *X*. **A, C, and E** Comparison of the mean values (± SEM) of the groups for plasma Hp(log10), eHSP27(log2), and endotoxin concentrations respectively. Significant differences are indicated by the asterisks above the period means. **B, D, and F** Within group comparisons for each group for plasma Hp(log10), eHSP27(log2), and endotoxin concentrations respectively across the five periods (only PreCh is labelled on the *x*-axis). The asterisks under the *x*-axis indicate statistically significant difference with the PreChallenge mean. ^+^*p* < 0.1; ^*^p < 0.05; ^**^p < 0.01; ^***^p < 0.001; ^****^p < 0.0001

There were significant effects of treatment (*p* = 0.0006) and period (*p* = 0.0002) on plasma Hp(log10) concentrations and a trend towards a treatment by period interaction (*p* = 0.065). The FRTN group had higher Hp(log10) concentrations relative to the PreChallenge mean through all periods, rising sharply during PENS then falling in Late PENS (Fig. 5A, B). Likewise, in PENS and Late PENS, the FRTN Hp(log10) mean concentrations were higher than those of the TC group. On the whole, the TC group mean concentration remained stable, and only tended to a rise in concentration during PENS (Fig. 5A, B). There was no effect of treatment (*p* = 0.192) for plasma eHSP27(log2) concentrations and a trend towards an effect of period (*p* = 0.0694). The mean eHSP27(log2) concentrations tended to drift lower with time (Fig. 5C, D). Mean plasma endotoxin levels showed no effects of treatment (*p* = 0.644) or period (*p* = 0.143; Fig. 5E, F).

### Interactions with rumen temperature and DMI during prechallenge, challenge, and recovery

In the same animals described in this study, strong negative linear relationships for thyroid stimulating hormone, T4, and adiponectin with rumen temperature were found (Wijffels et al. 2023). The FRTN group uniquely showed positive linear relationships between rumen temperature and leptin, and DMI and leptin. In contrast, the TC group displayed a positive linear relationship between DMI and adiponectin. Moreover, linear and elliptical relationships were found between rumen temperature and physiological and performance measures (Sullivan et al. 2022). Following these examples, the relationships of blood cell counts and the concentration of the inflammatory markers with rumen temperature and DMI were explored. The caveat in these explorations is the limited range in movement of rumen temperature for each treatment group whilst in the CCR, 39.22–39.80 °C for the FRTN group and a range of 39.54–40.65 °C was experienced by the TC group.

**Fig. 6:**
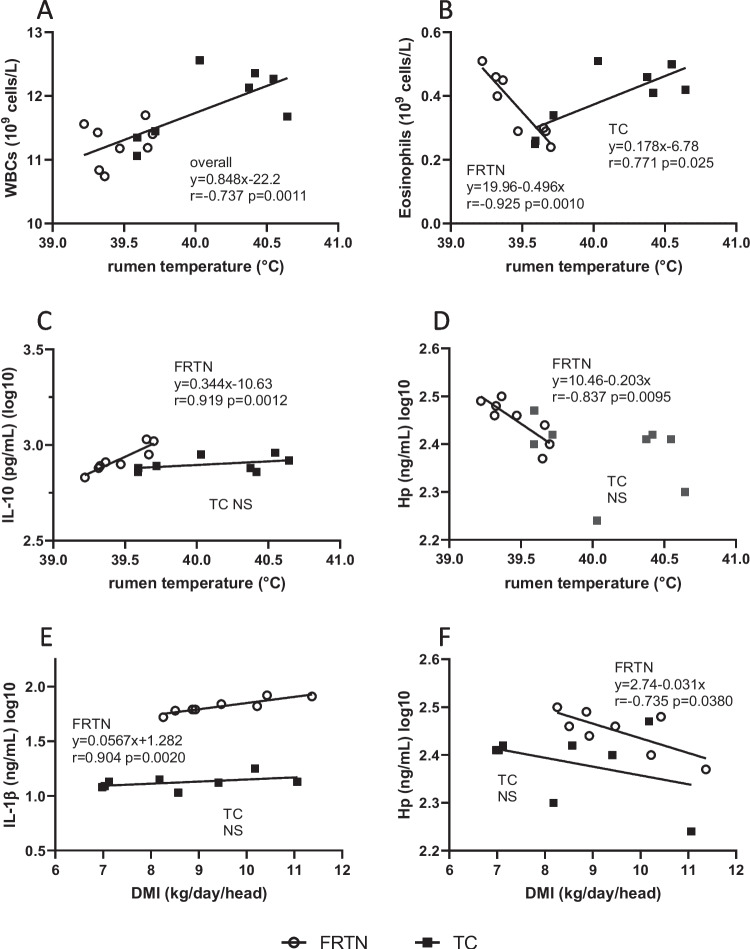
Linear relationships between daily mean cell counts and cytokine concentrations with daily mean rumen temperature or DMI of the thermally challenged (TC) and feed-restricted thermoneutral (FRTN) groups during the three periods in the CCR. **A** WBC count vs rumen temperature. **B** Eosinophil count vs rumen temperature. **C** IL-10(log10) concentration vs rumen temperature. **D** Hp(log10) concentration vs rumen temperature. **E** IL-1β(log10) concentration vs DMI. **F** Hp(log10) concentration vs DMI. The line-of-best fit and linear equation are given for the data pooled from both treatment groups (overall) or for each treatment group (TC and FRTN) along with the Pearson correlation *r* and level of significance. NS, not significant

Overall daily mean WBC count possessed a moderate positive relationship with rumen temperature (*r* = 0.737; *p* = 0.0011; Fig. 6A). With a 1 °C rise in rumen temperature, the WBC count rose by 0.85 × 10^9^ cell/L. Daily mean eosinophil counts showed strong negative correlation with rumen temperature (*r* =  − 0.925; *p* = 0.0010; Fig. 6B) for the FRTN group only. A 0.5 °C decrease in rumen temperature in these thermoneutral feed restricted animals was associated with a 100% increase in circulating eosinophils. Contrarily, the TC daily mean eosinophil counts displayed a moderate positive relationship with rumen temperature (*r* = 0.771; *p* = 0.025) which prescribed an 80% increment in eosinophil count as rumen temperature rose by 1 °C. None of the daily mean cell counts of the remaining leucocyte classes showed any interaction with daily mean rumen temperature or daily mean DMI.

Of the cytokines and inflammatory markers, only IL-10 and haptoglobin concentrations were found to have any relationship with rumen temperature, and those relationships were restricted to the FRTN group. Mean daily IL-10(log10) concentration was strongly and positively correlated with daily mean rumen temperature (*r* = 0.919; *p* = 0.0012; Fig. 6C). On the other hand, the relationship between daily mean haptoglobin(log10) concentrations and mean daily rumen temperature was strongly and negatively correlated (*r* =  − 0.837; *p* = 0.0095; Fig. 6D). Haptoglobin(log10) concentrations were also showed a moderate negative correlation with DMI (*r* = -0.735; *p* = 0.038; Fig. 6F). By contrast, no such response was elicited in the TC steers for IL-10 or haptoglobin concentrations as mean daily rumen temperature rose to 40.64 °C during Challenge and fell to 39.59 °C in Recovery. The mean daily IL-1β(log10) concentrations of the FRTN group demonstrated a strong positive relationship with mean daily DMI (*r* = 0.904; *p* = 0.0020; Fig. 6E). The mean daily IL-1β(log10) concentrations of the TC group was unresponsive to DMI.

## Discussion

This report is the final instalment of a series of four papers describing a comprehensive study of the responses of feedlot cattle during a moderate thermal challenge and through recovery. The responses are contrasted to those of feed-restricted thermoneutral animals (FRTN). This comparison is critical to discern the consequences of heat stress from those seen due to a sudden reduction in feed intake and subsequent realimentation (refeeding) in recovery. Descriptions of the performance and physiological responses, as well as the metabolic and metabolic hormone responses, have been reported elsewhere (Sullivan et al. 2022; Wijffels et al. 2022; 2023). The thermally challenged (TC) group was subjected to conditions typical of a moderate heatwave experienced over summer by feedlot cattle in southern Queensland, Australia. Overall, the metabolic and metabolic hormone trajectories for both groups differed revealing the different homeorhetic mechanisms applied to adjusting to new conditions. During Recovery and PENS, there was a return to a state similar although not identical to that of the PreChallenge state. In the current contribution, a survey of actors within the inflammatory system informed of the extent that the inflammation is invoked (or not) by a moderate heat load and the transience or persistence of those responses.

### Leucocyte counts

The cell counts and distribution of the blood leucocytes were investigated to assess the impact of moderate heat load in grain fed steers. The neutrophil count and the NL ratio best discriminated between the two treatment groups. For the FRTN group, the neutrophil counts were reduced during Challenge. The consequences of feed restriction on circulating leucocyte populations and subsets in ruminants are sparse. Kvidera et al. (2017a) reported no effects on leucocyte counts in mid-lactation Holsteins following 5 days of feed restriction at 60 and 80% of ad lib intake. However, reduced feeding of rats and dogs to 50–75% of normal intake for 7–14 days was associated with a downward trend in total WBC and neutrophil counts and reduced cellularity in bone marrow (Levin et al. 1993; Moriyama et al. 2008; Morita et al. 2017). Furthermore, short- and long-term caloric restrictions of humans and in rodent models have consistently shown reduced numbers of peripheral leucocytes and relocation of some subpopulations to the bone marrow (reviewed Lee and Dixit 2020). It has been proposed that the bone marrow and adipose tissue are refuges for some immune cell populations when a metabolic stress is imposed. It is possible that gradual underfeeding of the FRTN group during Challenge provoked these mechanisms. Overall, there was a strong positive linear relationship between WBC and rumen temperature. This relationship reflected the lowered WBC counts in the FRTN group whilst feed restricted rather than an overt rise in the TC group. It was notable that WBC, neutrophil, and lymphocyte counts of the TC group remained stable in Challenge suggesting an inability of leucocyte populations to respond to the reduced feed intake in a similar manner. A mechanism that could contribute to the maintenance of neutrophils in circulation may be their reduced clearance. The liver, spleen, and bone marrow are the major sites for removal of neutrophils from circulation (reviewed Strydom and Rankin 2013), and the reduced blood flow to these organs may have reduced the uptake of neutrophils.

In PENS, the neutrophil count of the TC group was markedly higher than that of the FRTN group, and this was obvious when inspecting the NL ratio when the TC group exhibited a comparatively high mean NL ratio. Interestingly, the elevated neutrophil count and NL ratio of the TC group in PENS coincides with peak levels of plasma activity of two liver enzymes, AST and GLDH (Wijffels et al. 2022). The increased release of these enzymes into plasma is highly suggestive of liver damage and/or repair. Plasma levels of the activities of these enzymes have been long used in medical and veterinary clinical biochemistry panels to assess liver involvement in disease. It is possible that the liver, other organs, and muscle in the TC steers are undergoing repair after a level of hypoxia due to the reduced blood flows to these sites as a response to increased thermal load (Hales 1973a,b). Such injury will cause the release of damaged-associated molecular patterns (DAMPS) and induce the expression and secretion of chemokines by the damaged cells and surrounding tissue. The consequences of this signalling are mobilisation of neutrophils from the bone marrow and their recruitment to damaged tissue (reviewed Strydom and Rankin 2013; Wang 2018). In Late PENS, the mean neutrophil counts and NL ratios of the groups converged and returned to equivalency with the PreChallenge mean.

The eosinophil has many of the same function as neutrophils in tissue damage and repair (reviewed Long et al. 2016; Abdala-Valencia et al. 2018). The eosinophil counts of the FRTN group showed some fluctuation across the periods. The eosinophil counts of the TC group were more variable across the periods and were significantly increased during PENS coinciding with the rise in neutrophil count. The differing behaviours returned opposing linear relationships with rumen temperature. As rumen temperature fell in the FRTN group, the eosinophil counts increased; in the TC group, the eosinophil counts increased as rumen temperature rose. Mean counts of the other two minor leucocyte subsets did not differ between treatments.

Generally, it appears that total and differential leucocyte counts are apparently not overly impacted by increased heat load in ruminants. The WBC counts of young bull calves were not affected by increasing THI, and PBL counts were variable but did not present a consistent trend (Kim et al. 2018). Others working with heifers or lactating cows under various thermal challenges did not report any consistent changes in leucocyte populations (Olbrich et al. 1972; Elvinger et al. 1992; Pandey et al. 2017; Jeelani et al. 2019). Only Kelley et al. (1982a), studying very young bull calves maintained at constant 35 °C for 14 days, saw increased lymphocyte counts and reduced neutrophil counts at day 7. The total WBC counts at 3, 7, and 14 days were unaffected by heat stress. These findings mostly concur with other studies of heat stressed growing ruminants.

It might be anticipated that leucocyte migration occurs into sites of local inflammation or hypoxia as a response to increased systemic heat load. However, it appears from the literature, and the current study that this does not occur. On the other hand, underfed animals do experience reduced total WBC, lymphocyte, and neutrophil counts, so the absence of these responses during increased heat load suggests altered regulation of the leucocyte trafficking.

### Cytokines

Whilst heat stroke is known to induce release of cytokines into the circulation (Leon 2006; Heled et al. 2013; Leon and Bouchama 2015), the responses of healthy animals during and after moderate heat stress, when animals are still capable of homeostatic and homeorhetic adjustments, are not clear. In the current study, the plasma TNFα and IL-1β concentrations were depressed in the TC group and remained so long after the thermal challenge. Interestingly, the FRTN group possessed a strong positive linear relationship with DMI and IL-1β concentrations suggestive that increased splanchnic blood flows and metabolic activity during realimentation may stimulate release of IL-1β into circulation. Increased circulating IL-1β concentrations and other cytokines upon refeeding have been reported in mouse and human models of fasting and short-term feed restriction (Petersen et al. 2014; McCoin et al. 2019; Speaker et al. 2016). There were no differences between the treatment groups for plasma IL-10 and IFNγ concentrations; the exception being that Recovery (realimentation) in the FRTN group was associated with a transient rise in IFNγ concentration. The FRTN group showed a positive linear relationship between IL-10 concentration and rumen temperature and modest positive relationship with DMI (*r* = 0.643; *p* = 0.083, data not shown).

Interestingly, the seven-day moderate thermal challenge employed here appeared to induce a similar response to the challenge imposed by chronic or seasonal heat stress in ruminants. Safa et al. (2018) monitored cooled and uncooled Holstein cows over summer in the 7 weeks post-partum. Although the mean rectal temperature of the uncooled cows was only 0.3 °C higher than that of the cooled cows, the plasma concentrations of TNFα and IL-1α were lower in the uncooled group for much of the 7-week period. Moreover, adaptation to increased heat load in indigenous goat breeds includes downregulated expression of a number of cytokine genes in the mesenteric lymph node (MLN) and the liver. Rashamol et al. (2019) and Madhusoodan et al. (2019) compared the cytokine expression in goats exposed to outdoor conditions through 45 days of summer with those of goats housed in a cooler indoor environment for the same period. There were moderate reductions in MLN expression of IL-18, TNFα, IFNβ, and IFNγ in the heat-stressed Malabari goats. Likewise, hepatic expression of IL-2, IL-6, IL-18, TNFα, and IFNβ was at least tenfold reduced in the heat stressed Black Salem goats. In a climate chamber environment, Koch et al. (2019) compared lactating Holsteins subjected to 4 days of moderate heat stress to their PFTN counterparts. They found that whilst IL-4 expression was approximately twofold increased in the jejunal mucosa relative to the PFTN group, expression of TNFα and IL-6 were not altered by heat stress.

Nonetheless, rises in plasma cytokines with increased heat load are also reported. 14-day heat stressed lambs (4–5-month age) exhibited increased plasma TNFα concentrations compared to counterparts housed with air conditioning, although IL-1 and IL-2 concentrations were not different between groups (Shi et al. 2020). Zhang et al. (2014) followed mid-lactation pastured Holstein cows over a summer season. The animals were blood sampled at the end of three consecutive 15–20-day periods of increasing mean daytime THI (ranging over 56–80). As daily mean rectal temperature increased with each period, plasma concentrations of TNFα and IL-10 rose also. It is probable that induction of an inflammatory response, whether local or systemic, during heat stress is dependent on many factors.

A tendency to some level of depression on the inflammatory system in heat stressed ruminants should not be unexpected, as a number of previous studies have revealed heat load suppression of in vivo immune functions and in vitro immune cell responses. At the cellular level, Do Amaral et al. (2011) found supressed phagocytic activity and oxidative capacity in blood neutrophils isolated from summer transition cows relative to cows that had received heat abatement. Mitogen-stimulated cell proliferation and IgM secretion by PBMCs collected from summer transition cows during an intense summer were significantly reduced compared to the responses of PBMCs isolated from spring cows (Lacetera et al. 2005). Using transcriptomics, Contreras-Jodar et al. (2018) revealed down-regulated expression of genes involved in immune cell proliferation and migration, and phagocytosis in the PBMC of lactating dairy goats after 35 days of constant diurnal cycling between 30–37 °C. At the whole animal level, Kelley et al. (1982b) observed significantly supressed delayed-type hypersensitivity reactions and contact sensitivity responses for 24–48 h following 7 or 14 days at constant 35 °C in very young bull calves (compared to calves maintained in thermoneutral conditions). Similar observations were reported when calves were exposed to alternating cycles of 12 h at 37 °C and 12 h at 32 °C for 5 and 12 days (Kelley et al. 1981).

### Haptogloblin (Hp)

Moderate heat stress did not affect the plasma haptoglobin (Hp) concentrations in the current study; in fact, the mean concentration of the TC group was remarkably stable for the duration of the experiment. Hp is a member of a subset of plasma proteins known as the acute phase proteins which include ceruloplasmin, serum amyloid A proteins, α1 acid glycoprotein, and many others (Ceciliani et al. 2012). Elevated levels of these proteins have been long associated with systemic responses to acute insults. Each of the acute phase proteins possess several roles in interacting with other proteins in plasma or at the cell membrane. Besides its role in protecting the oxidative status of the heme moiety of haemoglobin, Hp can bind directly with neutrophils to modulate behaviour and act as an extracellular chaperonin (Ceciliani et al. 2012).

Plasma Hp levels have been assessed, although not extensively, in bovine studies of feed restriction and in heat stress. In agreement with the results herein where the FRTN steers were subjected to ~ 20% mean reduction in DMI during Challenge, Kvidera et al. (2017a) found no significant change in plasma Hp concentrations in lactating cow after 20 and 40% reduction in DMI for 5 days relative to the ad lib period. Nor was there a Hp response by day 22 of 50% feed restriction in goats (Lérias et al. 2015). Reports of Hp responses in heat stressed ruminants are mixed. Kim et al. (2018) subjected groups of 6-month-old calves to three days at TN followed by 4 days of increased thermal load over a range of increasing THI (70–88). Plasma Hp concentration showed no consistent trend with THI. However, lactating goats subjected to 7 days heat stress in climate-controlled chambers showed a 30% rise in plasma Hp concentration compared to thermoneutral (but not pair fed) controls (Hamzaoui et al. 2013). Likewise, unshaded goats in hot conditions had a twofold rise within 7 days compared to their shaded control, and this situation persisted over the following 4 weeks (Al-Dawood 2017).

Curiously, the FRTN group plasma Hp concentration rose in the PENS and Late PENS periods whereas the TC group’s Hp levels remained stable. The Hp concentrations in the FRTN group showed strong negative correlations with rumen temperature and a modest negative correlation with DMI. Strictly, plasma Hp has not been measured during realimentation, so it is unknown if this is typical of compensatory gain. The higher Hp levels in the FRTN group did not appear to be associated with any other parameter investigated in this study, but it is interesting to note that the TC group did not respond likewise.

### Extracellular heat shock protein 27 (eHSP27)

Under the moderate thermal challenge delivered in the current study, plasma eHSP27(log2) concentrations were stable in the TC group, and paralleled that of the FRTN group. The families of HSPs (HSP90, HSP70, HSP60, HSP27 and HSP10) are abundantly expressed as intracellular proteins in most cells of most tissues. The term, eHSPs refers to extracellular HSPs, such as those detected in plasma and other body fluids. Lacking the signal sequence required for classical cellular secretion, the mode of release of eHSPs by viable, intact and non-apoptotic cells is unknown (Giuliano et al. 2011). Whilst the extracellular roles of eHSPs are not fully elucidated, they are suspected to participate in the innate immune response, tissue- or organ-wide signalling and cell proliferation, and to modulate the adaptative immune responses (reviewed Giuliano et al. 2011; Pockley and Henderson 2017). Much of the focus on eHSPs in ruminant thermal biology has been on eHSP70, and overall, circulating levels of eHSP70 appear to be associated with increased heat load in ruminants (Gaughan et al. 2013; Kim et al. 2018; Shilja et al. 2016). Consequently, an association between eHSP27 and heat stress in feedlot steers was investigated.

In the current study, no alterations in plasma eHSP27 were detected as a response to moderate thermal challenge nor to underfeeding and realimentation. Whilst the intracellular function of HSP27 appears to be cytoskeletal maintenance, eHSP27 has been implicated in suppression of inflammatory and immune system functions (Giuliano et al. 2011; van Noort et al. 2012). More specifically, eHSP27 has been implicated in vascular inflammation and ischemic reperfusion (Batulan et al. 2016). Given the blood flow and vascular changes which occur in response to increased core temperature, eHSP27 may be a good indicator of altered inflammatory state during heat stress. Baek et al. (2019) detected significant 30% increases in plasma eHSP27 in steers heat stressed for 3 or 6 days at constant 35 °C (60% RH). In outdoor natural conditions, plasma eHSP27 concentrations were ~ 60% elevated in lactating cows in summer relative to winter (Min et al. 2015). If eHSP27 can act as a reporter of vascular inflammation, there was clearly insufficient perturbation to the vasculature to incite release of eHSP27 in the moderately heat stressed or feed restricted steers in the current study.

### Endotoxin

FRTN and TC groups did not experience endotoxemia in any period throughout the experiment. Mean plasma endotoxin levels were not higher than 0.75 EU/mL and well within the interquartile range of 0.23–3.89 EU/mL for endotoxin concentration in plasma of healthy humans (Gnauck et al. 2016). Endotoxin, also known as lipopolysaccharide (LPS), is a glycolipid component of the outer cell membrane of Gram-negative bacteria which includes the Enterobacteriaceae. Endotoxemia is posited as an indirect consequence of heat stress in mammals and a direct consequence of the loss of gut integrity. Rat models of heat stroke have provided evidence of splanchnic hypoxia, increased gut permeability, and rising portal LPS concentration (Hall et al. 1999; 2001). Studies of production pigs subjected to acute hyperthermia (in the absence of diurnal temperature cycling) also provided evidence of altered morphology of the intestinal microvilli and epithelial morphology, reduced intestinal integrity, impaired tight junction structure, altered expression of tight junction proteins, and the appearance of endotoxin in plasma (Pearce et al. 2013; 2014; reviewed Gabler and Pearce 2015). Circulating endotoxin, regardless of how it arises, is known to induce profound inflammatory responses in all mammals, including ruminants (Mani et al. 2012).

The evidence for a similar series of events in heat stressed ruminants may not be as clear cut. Koch et al. (2019) and Eslamizad et al. (2020) looked for evidence of compromised gut integrity and inflammatory consequences in lactating Holsteins subjected to 4 days of moderate heat stress in climate chambers. Careful study of the jejunal mucosa and submucosa revealed the infiltration of novel macrophage-like cells in response to heat stress, but no changes to the mucosa could be observed (Koch et al. 2019). Similarly, Eslamizad et al. (2020) found no differences in the expression of components of inflammatory signalling at both the protein and transcript levels by rumen papillae collected from heat stressed cows and their FRTN counterparts. Furthermore, apparent gut responses to heat stress needs to be carefully differentiated from the impact on the intestinal and ruminal mucosae of reduced feed intake alone (Zhang et al. 2013a,b; Kvidera et al. 2017a,b).

## Concluding comments

The inflammatory status of grain fed feedlot cattle during a moderate heat load and subsequent recovery was examined in this study. There were no indications of provocation of a systemic inflammatory or stress response, nor endotoxemia as an endpoint indicator of impaired gut integrity relative to FRTN counterparts. Two findings were indicative of responses in the TC steers that differed from their FRTN counterparts. Firstly, it was notably that during Challenge, the movement of WBC and neutrophil numbers relative to PreChallenge was greater in the FRTN group than the TC group. In the latter, there was absence of changes in numbers of circulating leucocytes. This may suggest an inability by the leucocytes of the TC group to respond appropriately, along with reduced uptake and clearance of neutrophils by the liver and spleen during Challenge. Secondly, the plasma concentrations of the two major proinflammatory cytokines, TNFα and IL-1β, were persistently reduced compared to the FRTN group. In healthy animals attempting to reduce endogenous heat production, down regulation of the inflammatory response at the systemic level seems appropriate. The previous reports on the endocrine and metabolic response of these thermally challenged steers showed homeorhetic adjustments to the increased heat load and subsequent adjustment after the thermal challenge to arrive at similar metabolic hormone milieu and metabolic state as their FRTN counterparts (Wijffels et al. 2023). Moreover, both the FRTN and TC steers had recovered and strongly increased DMI in PENS and Later PENS, and experienced rapid live weight gain; the treatment groups could not be differentiated on these performance measures. It is intriguing then that the TNFα and IL-1β concentrations of the TC steers were persistently lower compared to the FRTN steers well into recovery and ‘finishing’ (i.e,. in PENS and Late PENS). Does this suggest that the moderately heat stressed steer will incur a greater energy cost in mounting a future inflammatory response? This potential consequence implies vulnerability in limiting the early phases of future viral and bacterial infections.

### Supplementary Information

Below is the link to the electronic supplementary material.

Supplementary file1 (DOCX 167 KB)
