# Supplementary material for: Comparing the responses of grain-fed feedlot cattle under moderate heat load and during subsequent recovery with those of feed-restricted thermoneutral counterparts: blood cells and inflammatory markers
Source: Int J Biometeorol. 2023 Dec 13;68(2):211–27. doi: 10.1007/s00484-023-02584-3 (PMC10794350; doi:10.1007/s00484-023-02584-3)
Supplement: Supplementary file 1 [file 484_2023_2584_MOESM1_ESM.docx]

Supplementary Table 1. ELISA reagents

Analyte	Capture Antibody	Supplier	Conc. (μg/mL)	Detection Antibody	Supplier	Conc. (μg/mL)
IL-1β	Rabbit anti-bovine IL1β	Serotec SEAHP851Z	1.0	Rabbit anti-bovine IL-1β Biotin	Serotec SEAHP851B	0.5
IL-10	Mouse monoclonal anti-IL10	Serotec MCA2110	0.2	Mouse monoclonal anti-IL-10 Biotin	Serotec MCA2111B	0.2
IFNγ	Mouse anti-bovine IFNγ	Serotec SEMCA2112	1.0	Mouse anti-bovine IFNγ Biotin	Serotec SEMCA1783B	0.25
TNFα	Goat anti-bovine TNFα	R&D Systems DY2279	0.5	Goat anti-bovine TNFα Biotin	R&D Systems DY2279	0.4
HSP27	Human HSP27 capture antibody	R&D Systems DuoSet DY1580	0.2	Human HSP27 detection antibody	R&D Systems DuoSet DY1580	0.1
Haptoglobin	Rabbit Anti-Human Haptoglobin Affinity purified	In-house	1.25	HRP Conjugated Anti-Human Haptoglobin	In-house	1.25

Supplementary Table 2. Assay performance (mean±SEM)

Analyte	LoB*	LoD*	Intra-assay %CV	Inter-assay %CV
TNFα (ng/mL)	2.6±0.6	3.4±0.9	3.0±0.2	12.3±1.6
IL-1β (ng/mL)	0.9±0.1	1.7±0.3	4.2±0.4	12.3±0.9
IFNγ (pg/mL)	9.0±1.25	12.3±2.0	3.3±0.0	10.1±1.8
IL-10 (pg/mL)	282.0±25.6	423.9±21.9	3.8±0.0	9.2±0.8
Hp (ng/mL)	88.7±46.0	163.5±55.1	3.6±0.1	9.8±1.0
HSP27 (pg/mL)	122±20	89±17	3.3±0.4	9.9±1.1

*The Limit of the Blank (LoB) and Limit of Detection (LoD) were determined using the method of Armbruster and Pry (2008).
